# Variability in Insurance Adequacy for Children With Special Health Care Needs on Medicaid

**DOI:** 10.1001/jamahealthforum.2025.5366

**Published:** 2025-12-05

**Authors:** Amy J. Houtrow, Matt Hall, James M. Perrin, Dennis Z. Kuo, Jeffrey D. Colvin, Allysa Ware, Jeffery S. Schiff, Jay Berry, Ryan J. Coller

**Affiliations:** 1 Department of Physical Medicine & Rehabilitation, University of Pittsburgh School of Medicine, Pittsburgh, Pennsylvania; 2 Children’s Hospital Association, Lenexa, Kansas; 3 MassGeneral Brigham for Children, Harvard Medical School, Boston, Massachusetts; 4 Department of Pediatrics, University of Rochester School of Medicine and Dentistry, Rochester, New York; 5 Department of Pediatrics, Children’s Mercy Hospital, University of Missouri–Kansas City School of Medicine, Kansas City; 6 Family Voices, Concord, Massachusetts; 7 Academy Health, Evidence-Informed State Health Policy Institute, Washington, DC; 8 Division of General Pediatrics, Boston Children’s Hospital, Harvard Medical School, Boston, Massachusetts; 9 Department of Pediatrics, University of Wisconsin School of Medicine and Public Health, Madison

## Abstract

This survey study quantifies state-to-state variation and predictors of Medicaid insurance continuity and adequacy among children with special health care needs.

## Introduction

Children with special health care needs (SHCN) with inadequate health insurance coverage have difficulty getting needed care, more preventable hospitalizations, and poorer health.^1,2^ Medicaid is the primary insurer for nearly one-half of US children with SHCN and the secondary insurer for some children with disabilities, but there is substantial variability across states.^3^ Quantifying state-to-state variation and predictors of Medicaid insurance continuity and adequacy among children with SHCN can inform states considering changes to Medicaid that may affect insurance continuity and adequacy, and therefore, health care utilization and health outcomes.

## Methods

We conducted an analysis of children with SHCN aged 0 to 18 years on Medicaid using the publicly available data from the 2022 and 2023 National Survey of Children’s Health (NSCH). We used survey weights to provide national and state-specific estimates. Medicaid insurance was considered continuous if the child was insured at the time of the survey and the prior 12 months without gaps. Medicaid insurance was considered adequate if it (1) usually or always met the child’s needs, (2) usually or always allowed the child to see needed health care professionals, and (3) had no out-of-pocket costs or out-of-pocket costs were usually or always reasonable.^4^ Percentages of continuous and adequate Medicaid insurance were reported by state. Using logistic regression, national estimates were adjusted for health conditions consistently and often greatly affected their daily activities (activity limitations) and sociodemographic characteristics. These variables were chosen due to their relationship to the presence of special health care needs and/or insurance coverage.^3,4^ The University of Pittsburgh Institutional Review Board considered this analysis as exempt non–human subjects research because the data are publicly available. We followed the STROBE reporting guideline. Analyses were conducted using SAS version 9.4 (SAS Institute). Data were analyzed from May to September 2025.

## Results

According to 2022-2023 NSCH data, Medicaid covered 6.6 million children with SHCN in the US, of whom 98% had continuous insurance and 80.8% had adequate insurance (Table). There were no differences in continuity of Medicaid insurance by sociodemographic characteristics, but children with SHCN with both Medicaid and commercial insurance reported lowest adjusted rates of adequate insurance (65.4%; 95% CI, 61.0-69.6), and children with SHCN with activity limitations less commonly had adequate insurance (74.3%; 95% CI, 69.5-78.6). The percentage of Medicaid-insured children with SHCN with continuous and adequate insurance varied by nearly 30 percentage points, from a low of 59.8% (95% CI, 47.8-71.9) in Illinois to a high of 88.7% (95% CI, 82.6-94.9) in Virginia (Figure).

**Table.** Unadjusted and Adjusted^a^ Percentages of Continuous and Adequate Health Insurance by Sociodemographic Characteristics Among Children With Special Health Care Needs (SHCN) Covered by Medicaid Insurance

Characteristic	Children with SHCN with continuous health insurance		Children with SHCN with adequate health insurance		Children with SHCN with continuous and adequate health insurance	
	Unadjusted, %	Adjusted, % (95% CI)	Unadjusted, %	Adjusted, % (95% CI)	Unadjusted, %	Adjusted, % (95% CI)
Total	98.0	NA	80.8	NA	79.7	NA
Sex						
Female	97.5	98.0 (96.4-98.9)	78.8	79.9 (76.9-82.7)	77.1	77.9 (74.4-81.2)
Male	98.4	98.9 (98.2-99.3)	82.3	83.5 (81.5-85.3)	81.6	82.8 (80.8-84.6)
Race and ethnicity^b^						
Asian	95.3	97.2 (90.4-99.2)	78.5	81.6 (77.6-85)	79.0	81.0 (78.6-83.1)
Hispanic	98.9	97.0 (94.2-98.5)	82.6	80.1 (70-87.4)	78.3	79.0 (74.1-83.1)
Non-Hispanic Black	98.9	99.2 (98.0-99.7)	79.7	82.9 (79.1-86.1)	82.3	82.7 (78.9-85.9)
Non-Hispanic White	96.2	98.9 (98.4-99.2)	80.9	81.9 (79.6-84.1)	77.1	78.6 (68.3-86.3)
Non-Hispanic other race	98.2	98.4 (96.9-99.2)	81.4	82.2 (77.8-85.8)	80.8	81.4 (77.0-85.1)
Family income as a percentage of FPL						
<100	97.2	97.8 (96.0-98.8)	84.9	84.1 (81.4-86.4)	83.1	82.3 (78.8-85.3)
100-199	98.4	98.9 (97.9-99.4)	79.9	79.9 (76.4-83.1)	79.0	79.2 (75.7-82.3)
200-399	98.1	98.6 (97.1-99.3)	79.1	82.0 (79.0-84.6)	78.0	80.9 (77.8-83.6)
≥400	99.4	99.5 (98.7-99.8)	71.8	80.6 (74.9-85.3)	71.6	80.0 (74.3-84.6)
Rurality						
Rural	97.9	98.9 (97.1-99.6)	79.9	84.9 (82.0-87.3)	83.5	83.9 (80.8-86.5)
Nonrural	98.7	98.5 (97.9-98.9)	84.3	81.4 (79.4-83.2)	78.7	80.1 (77.9-82.1)
Nativity						
Child born in US	98.1	98.6 (98.1-99.0)	80.8	81.9 (80.2-83.5)	79.6	80.7 (78.9-82.5)
Child born outside US	92.9	95.9 (85.3-98.9)	81.7	85.7 (70.1-93.9)	79.7	84.0 (69.0-92.5)
Activity limitations^c^						
No	98.1	98.6 (98.1-99.0)	82.8	83.6 (81.8-85.2)	81.5	82.3 (80.4-84.1)
Yes	97.5	98.2 (96.1-99.1)	72.5	74.3 (69.5-78.6)	71.6	73.2 (68.4-77.5)
Medicaid coverage						
Medicaid only	97.0	98.5 (98.1-98.9)	85.1	85 (83.3-86.6)	83.7	83.8 (81.9-85.5)
Private insurance plus Medicaid	98.5	98.5 (98.1-98.9)	63.6	65.4 (61.0-69.6)	63.2	64.6 (60.2-68.8)

Abbreviations: NA, not applicable; FPL, federal poverty level.

^a^ Adjusted for sex, race and ethnicity, family income as measured by FPL, rurality, nativity, activity limitations, and presence of concurrent private insurance.

^b^ Race and ethnicity were reported by respondents. The other race category includes children identified as American Indian or Alaska Native, Native Hawaiian or Other Pacific Islander, and multirace due to small sample size in many states.

^c^ Conditions that consistently and often greatly affect their daily activity.

**Figure.**
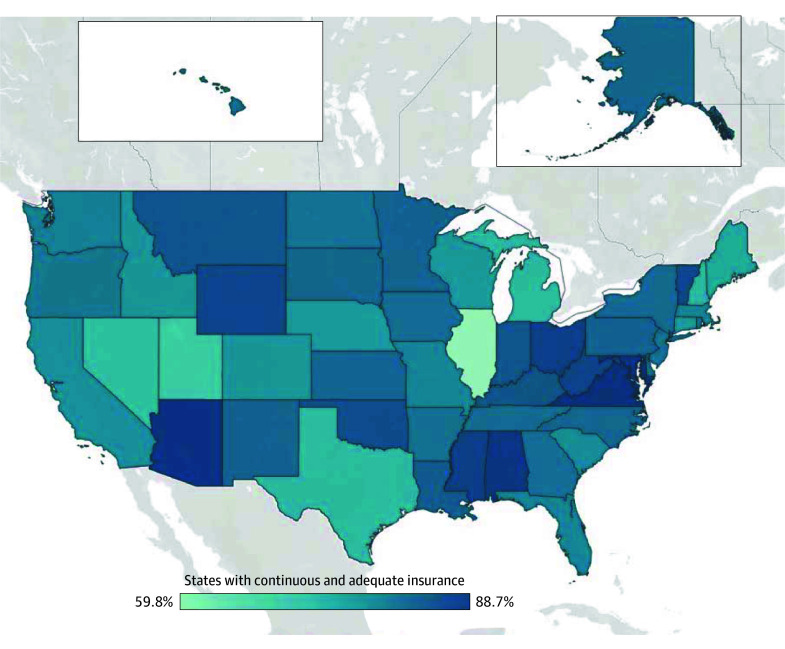
US Map of Children With Special Health Care Needs Covered by Medicaid With Continuous and Adequate Health Insurance by State US map demonstrating the variability in continuous and adequate insurance among Medicaid-insured children with special health care needs. Illinois had the lowest percentage of continuous and adequate Medicaid insurance (59.8%; 95% CI, 47.8-71.9), while Virginia had the highest (88.7%; 95% CI, 82.6-94.9). Across the country, 5 221 879 of 6 555 345 Medicaid-insured children with special health care needs (79.7%) had continuous and adequate insurance.

## Discussion

To our knowledge, this study is the first to describe Medicaid continuity and adequacy among children with SHCN and report wide state-to-state variation from contemporary NSCH data. We found that 80% of Medicaid-insured children with SHCN had continuous and adequate insurance, markedly higher than the 66% of commercially insured children with SHCN.^1^ Because Medicaid is a state-federal partnership, states’ Medicaid program vary and likely impact the state-to-state variability found here. Given demonstrated associations between inadequate insurance and worse children with SHCN health outcomes,^2,5^ states should prioritize policies promoting continuous and adequate insurance coverage for this vulnerable population, especially those states with lower percentages of insurance continuity and adequacy. Medicaid changes that result in inadequate care or dropped coverage could exacerbate challenges to achieving adequate access to health care services and worsen health outcomes for children with SHCN.^2,5^ Importantly, these identified disparities indicate the need to improve Medicaid continuity and adequacy for children with SHCN with activity limitations and those who are dually covered who tend to have extensive health care needs.^1,4,6^ This nationally representative cross-sectional study is limited by reliance on parental or caregiver recall and cannot be used for causal inferences. Nonetheless, tracking these data over time would provide an important foundation on which to assess the impact of state and federal policy on health outcomes for this key group of children and youth.

## Notes

Supplement. Data Sharing Statement.

## References

[1] Validova A, Strane D, Matone M, . Underinsurance among children with special health care needs in the United States. JAMA Netw Open. 2023;6(12):e2348890. doi:10.1001/jamanetworkopen.2023.48890 38147335 PMC10751585

[2] Brantley E, Ku L. Continuous eligibility for Medicaid associated with improved child health outcomes. Med Care Res Rev. 2022;79(3):404-413. doi:10.1177/10775587211021172 34525877

[3] Medicaid and CHIP Payment and Access Commission. Access in Brief: children and youth with special health care needs. Accessed May 4, 2025. https://www.macpac.gov/wp-content/uploads/2024/08/Access-in-Brief-Children-and-Youth-with-Special-Health-Care-Needs.pdf

[4] Yu J, Perrin JM, Hagerman T, Houtrow AJ. Underinsurance among children in the United States. Pediatrics. 2022;149(1):e2021050353. doi:10.1542/peds.2021-050353 34866156 PMC9647940

[5] Flores G, Lin H, Walker C, . The health and healthcare impact of providing insurance coverage to uninsured children: a prospective observational study. BMC Public Health. 2017;17(1):553. doi:10.1186/s12889-017-4363-z 28592269 PMC5463460

[6] Berry JG, Perrin JM, Hoover C, Rodean J, Agrawal RK, Kuhlthau KA. Health care insurance adequacy for children and youth with special health care needs. Pediatrics. 2021;148(4):e2020039891. doi:10.1542/peds.2020-039891 34535570

