# Supplementary material for: Variability in Insurance Adequacy for Children With Special Health Care Needs on Medicaid
Source: JAMA Health Forum. 2025 Dec 5;6(12):e255366. doi: 10.1001/jamahealthforum.2025.5366 (PMC12681037; doi:10.1001/jamahealthforum.2025.5366)
Supplement: Supplement. — Data Sharing Statement. [file jamahealthforum-e255366-s001.pdf]

Data Sharing Statement

Houtrow. Variability in Insurance Adequacy for Children With Special Health Care Needs on Medicaid. *JAMA Health Forum*. Published December 05, 2025.
doi:10.1001/jamahealthforum.2025.5366

Data
Data available: Yes
Data types: Other (please specify)
Additional Information: The data is publicly available
How to access data: https://www.childhealthdata.org
When available: beginning date: 01-01-2016

Supporting Documents
Document types: None

Additional Information
Who can access the data: please do not request data from authors. it is already available to the public.
Types of analyses: There is analyses available at https://www.childhealthdata.org
Mechanisms of data availability: available to public through website by the sponsor of the NSCH
Any additional restrictions: n/a
